# Supplementary material for: Role of the V2R–βarrestin–Gβγ complex in promoting G protein translocation to endosomes
Source: Commun Biol. 2024 Jul 7;7:826. doi: 10.1038/s42003-024-06512-y (PMC11228049; doi:10.1038/s42003-024-06512-y)
Supplement: Supplementary file 2 — Supplementary information [file 42003_2024_6512_MOESM2_ESM.pdf]

Role of the V2R–βarrestin–Gβγ complex in promoting G protein translocation to endosomes

Badr Sokrat[1,2#†], Anthony H. Nguyen[3#], Alex R. B. Thomsen[3,4†], Li-Yin Huang[3], Hiroyuki Kobayashi[2], Alem W. Kahsai[4], Jihee Kim[3], Bing X. Ho[3], Symon Ma[3], John Little IV[3], Catherine Ehrhart[3], Ian Pyne[3], Emmery Hammond[3], Michel Bouvier[1,2]*

[1] Department of Biochemistry and Molecular Medicine, University of Montreal, Montreal, Quebec, H3T 1J4 Canada

[2] Institute for Research in Immunology and Cancer, University of Montreal, Montreal, Quebec, H3T 1J4 Canada

[3] Department of Biochemistry, Duke University School of Medicine, Durham, NC 27710, USA

[4] Department of Medicine, Duke University Medical Center, Durham, NC 27710, USA

# These authors contributed equally to the manuscript

† Present address: Department of Molecular Pathobiology, New York University School of Dentistry, New York, NY 10010

* Corresponding author

e-mail address: michel.bouvier@umontreal.ca

Supplementary Figures

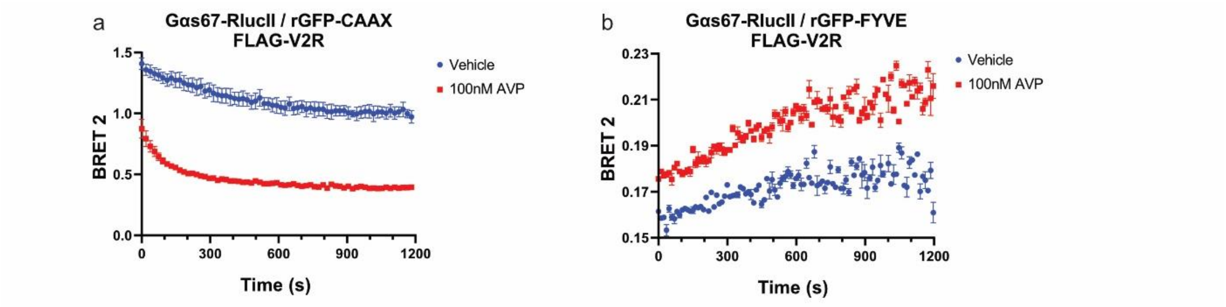

Figure S1: Kinetics of Gα_s trafficking from the plasma membrane to the endosomes. a) AVP-induced (100nM) Gα_s dissociation from the plasma membrane monitored by ebBRET between Gα_s67-RlucII and rGFP-CAAX in HEK293 cells. **b)** AVP-induced (100nM) Gα_s trafficking to the endosomes stimulation monitored by ebBRET between Gα_s67-RlucII and rGFP-FYVE in HEK293 cells. Data are represented as the mean +/– SEM (n=4) (data in supplementary files).

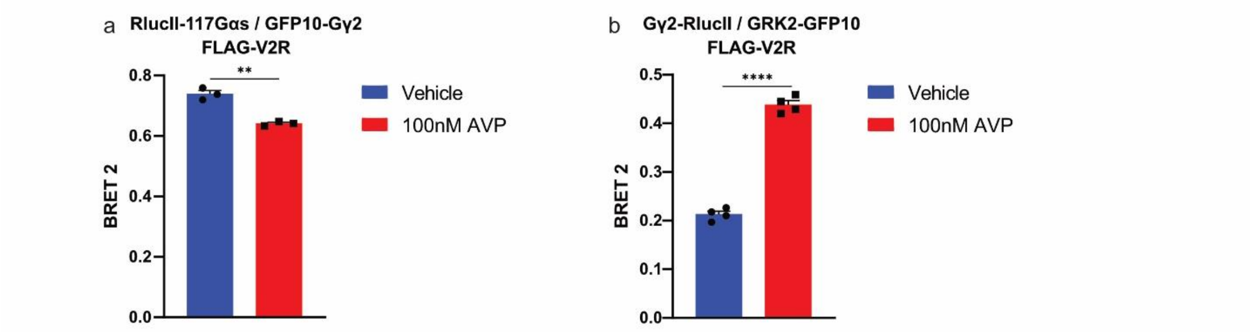

Figure S2: Gα and Gβγ dissociation after V2R activation. a) AVP-induced (100nM) dissociation of RlucII-117Gα_s and GFP10-Gγ2 after 20 min stimulation monitored by BRET in HEK293 cells. **b)** AVP-induced (100nM) recruitment of GRK2-GFP10 by Gγ2-RlucII after 20 min stimulation monitored by BRET in HEK293 cells. Data are represented as the mean +/– SEM (n=3-4) and statistical significance was assessed using an unpaired t test (** $P \leq 0.01$; **** $P \leq 0.0001$).

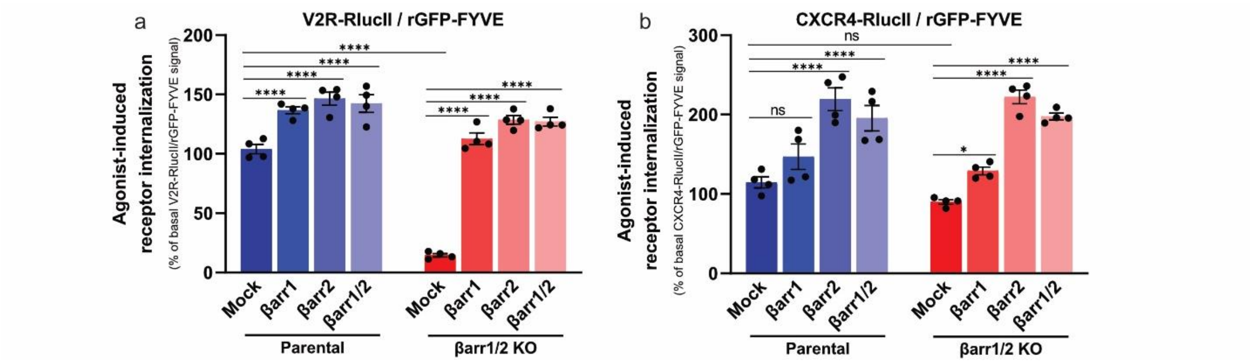

Figure S3: Role of βarr in V2R and CRXR4 endocytosis. a) V_2R endocytosis is monitored by ebBRET using V_2R-RlucII and rGFP-FYVE in parental HEK293SL cells and βarr1and βarr2 KO (βarr1/2 KO) cells with or without βarr1 and βarr2 supplementation after 20 min 100nM AVP stimulation. **b)** CXCR4 endocytosis is monitored by ebBRET using CXCR4-RlucII and rGFP-FYVE in parental HEK293SL cells and βarr1/2 KO cells with or without βarr1 and βarr2 supplementation after 20 min 100nM CXCL12 stimulation. Data are represented as the mean +/– SEM (n=4) and statistical significance of the differences was assessed using a two-way ANOVA followed by Holm-Šídák's multiple comparison test (ns nonsignificant; * $P \leq 0.05$; **** $P \leq 0.0001$).

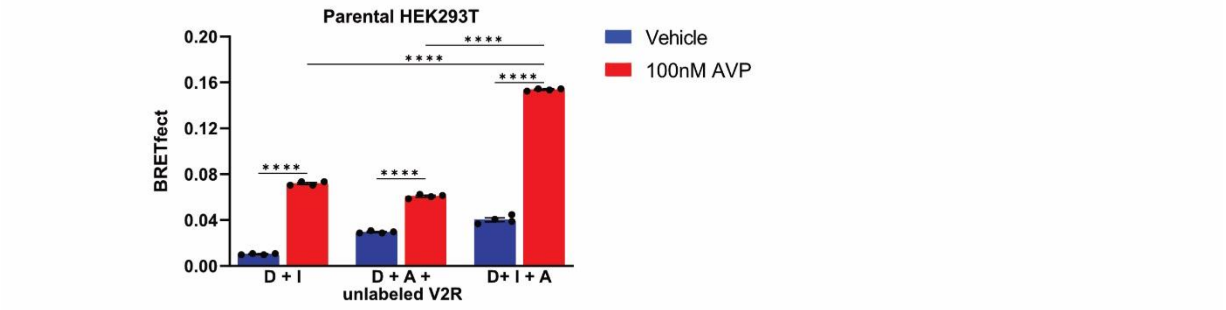

Figure S4: V_2R–βarr2–Gβγ complex formation monitored by BRETfect assay. Co-expression of BRETfect constructs (RlucII donor (D) fused to βarr2, mTFP intermediate (I) fused to V_2R and energy acceptor YFP (A) fused to $G\gamma_2$) in parental HEK293T followed by vehicle or 100nM AVP stimulation for 20 min. Unlabeled V_2R is co-transfected with the Donor (RlucII fused to βarr2) + the Acceptor (YFP fused to $G\gamma_2$). Data are represented as the mean +/– SEM (n=4) and statistical significance of the differences was assessed using a two-way ANOVA followed by Holm-Šídák's multiple comparison test (**** $P \leq 0.0001$).

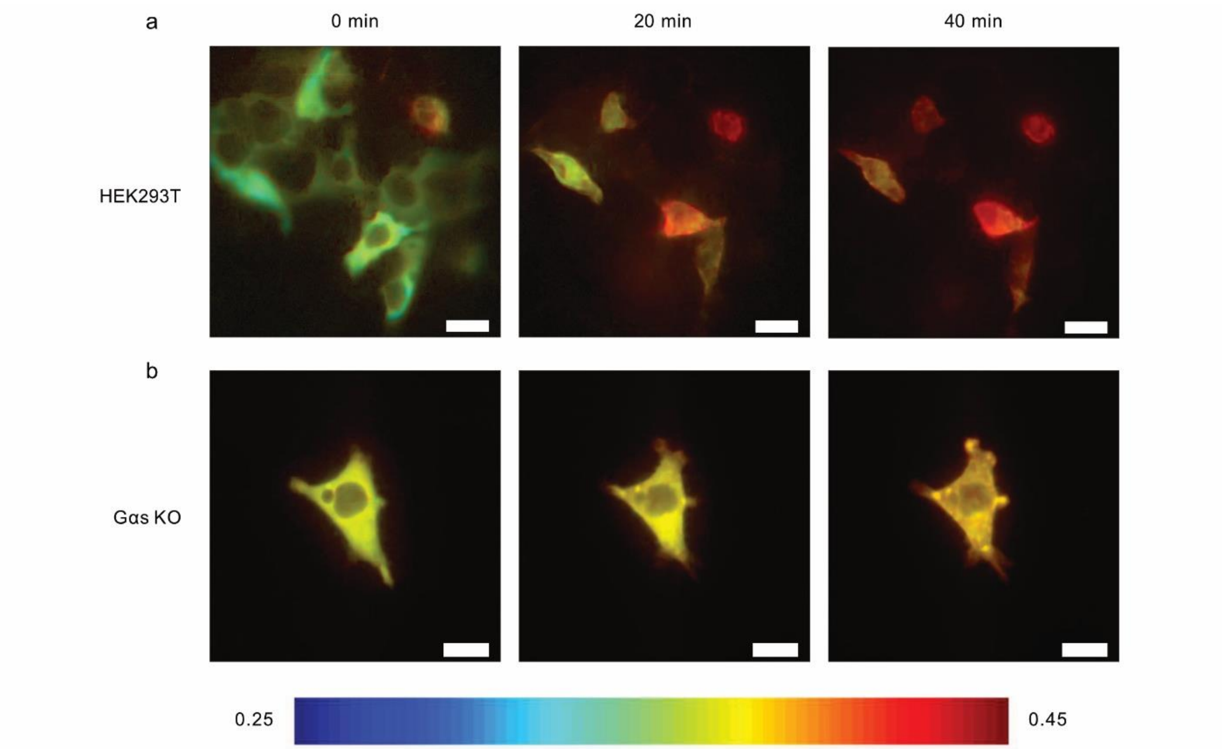

Figure S5: V_2R–βarr2–Gβγ complex formation monitored by BRETfect microscopy in parental HEK293T and Gα_S KO cells. a-b) Co-expression of βarr2-RlucII, V_2R-mTFP and Gγ_2-YFP in parental HEK293T cells (**a**) or in total Gα KO cells (**b**) followed by 100nM AVP stimulation and image acquisition by luminescence microscopy. The numeric scale of the heat-map legend represents calculated BRET ratios. Scale bar: 5μm.

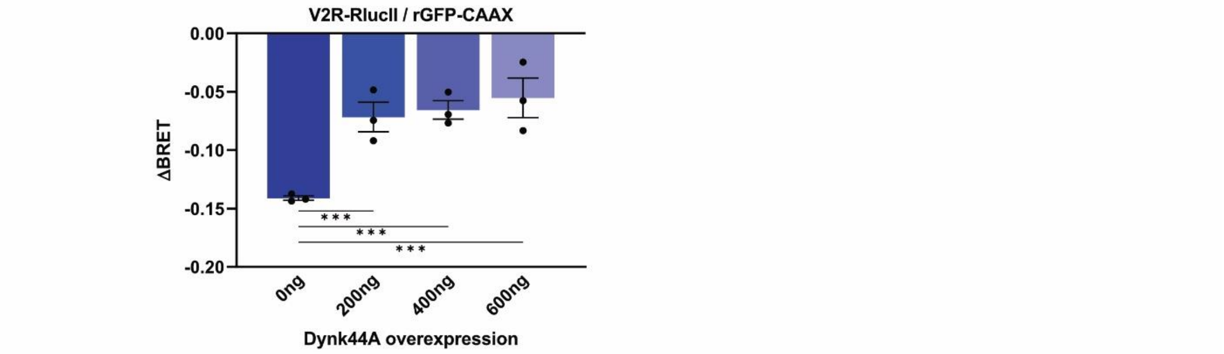

Figure S6: V_2R internalization in inhibited by DynK44A. Receptor internalization in HEK293SL cells is monitored by ebBRET between V_2R-RlucII and rGFP-CAAX with co-expression of increasing amounts of DynK44A. Data are represented as the mean +/– SEM (n=3) and statistical significance of the difference was assessed using a two-way ANOVA followed by Holm-Šídák's multiple comparison test (*** $P \leq 0.001$).

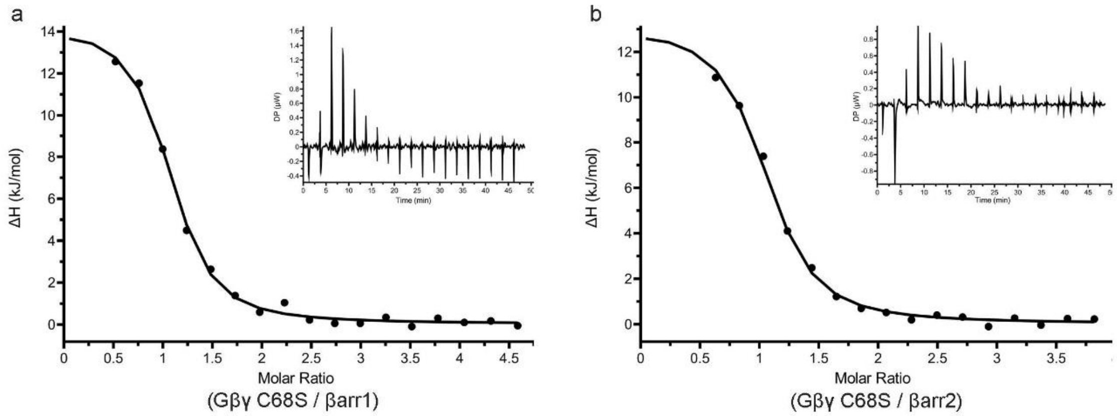

Figure S7: Gβγ C68S maintains ability to bind to inactive βarr1/2. Binding isotherm and thermogram (inset) between Gβγ with a C68S mutation on $G\gamma_2$ and **a)** inactive βarr1 ($K_D = 0.8 \pm 0.1$ µM; N = 1.0 site), and **b)** inactive βarr2 ($K_D = 0.9 \pm 0.1$ µM; N = 1.0 site). Both binding events (in **a** and **b**) appear to have an additional early binding site, which were excluded, and the experiment was fitted to a one-site binding model.
