# Supplementary material for: Role of the V2R–βarrestin–Gβγ complex in promoting G protein translocation to endosomes
Source: Commun Biol. 2024 Jul 7;7:826. doi: 10.1038/s42003-024-06512-y (PMC11228049; doi:10.1038/s42003-024-06512-y)
Supplement: Supplementary file 3 — Description of Additional Supplementary Files [file 42003_2024_6512_MOESM3_ESM.pdf]

Description of Additional Supplementary Files

File name: Supplementary Data

Description: The source data behind the graphs in the paper

File name: Supplementary movies 1-2.

Description: Movies of microscopy
