# Supplementary material for: Role of the V2R–βarrestin–Gβγ complex in promoting G protein translocation to endosomes
Source: Commun Biol. 2024 Jul 7;7:826. doi: 10.1038/s42003-024-06512-y (PMC11228049; doi:10.1038/s42003-024-06512-y)
Supplement: Supplementary file 7 — Reporting Summary [file 42003_2024_6512_MOESM7_ESM.pdf]

nature portfolio

Corresponding author(s): Michel Bouvier

Last updated by author(s): May 10, 2024

Reporting Summary

Nature Portfolio wishes to improve the reproducibility of the work that we publish. This form provides structure for consistency and transparency in reporting. For further information on Nature Portfolio policies, see our Editorial Policies and the Editorial Policy Checklist.

Statistics

For all statistical analyses, confirm that the following items are present in the figure legend, table legend, main text, or Methods section.

n/a	Confirmed	
☐	☒	The exact sample size (*n*) for each experimental group/condition, given as a discrete number and unit of measurement
☐	☒	A statement on whether measurements were taken from distinct samples or whether the same sample was measured repeatedly
☐	☒	The statistical test(s) used AND whether they are one- or two-sided<br>*Only common tests should be described solely by name; describe more complex techniques in the Methods section.*
☒	☐	A description of all covariates tested
☒	☐	A description of any assumptions or corrections, such as tests of normality and adjustment for multiple comparisons
☐	☒	A full description of the statistical parameters including central tendency (e.g. means) or other basic estimates (e.g. regression coefficient) AND variation (e.g. standard deviation) or associated estimates of uncertainty (e.g. confidence intervals)
☒	☐	For null hypothesis testing, the test statistic (e.g. F, t, r) with confidence intervals, effect sizes, degrees of freedom and P value noted<br>*Give P values as exact values whenever suitable.*
☒	☐	For Bayesian analysis, information on the choice of priors and Markov chain Monte Carlo settings
☒	☐	For hierarchical and complex designs, identification of the appropriate level for tests and full reporting of outcomes
☒	☐	Estimates of effect sizes (e.g. Cohen's *d*, Pearson's *r*), indicating how they were calculated

Our web collection on statistics for biologists contains articles on many of the points above.

Software and code

Policy information about availability of computer code

Data collection	BRET and BRETfect were measured on a Mithras LB940 photon-counting plate reader (Berthold Technologies) and a Spark multimode microplate reader (Tecan). ITC measurements were performed using the MicroCal PEAQ-ITC (Malvern Panalytical).
Data analysis	Microplate assays were analysed using GraphPad Prism. ITC Data was analyzed using the MicroCal PEAQ-ITC analysis software (version 1.41). Image quantification was performed using ImageJ.

For manuscripts utilizing custom algorithms or software that are central to the research but not yet described in published literature, software must be made available to editors and reviewers. We strongly encourage code deposition in a community repository (e.g. GitHub). See the Nature Portfolio guidelines for submitting code & software for further information.

Data

Policy information about availability of data

All manuscripts must include a data availability statement. This statement should provide the following information, where applicable:

- Accession codes, unique identifiers, or web links for publicly available datasets
- A description of any restrictions on data availability
- For clinical datasets or third party data, please ensure that the statement adheres to our policy

All data, associated methods, and sources of materials are available in the main text or in the supporting information.

Research involving human participants, their data, or biological material

Policy information about studies with human participants or human data. See also policy information about sex, gender (identity/presentation), and sexual orientation and race, ethnicity and racism.

Reporting on sex and gender	n/a
Reporting on race, ethnicity, or other socially relevant groupings	n/a
Population characteristics	n/a
Recruitment	n/a
Ethics oversight	n/a

Note that full information on the approval of the study protocol must also be provided in the manuscript.

Field-specific reporting

Please select the one below that is the best fit for your research. If you are not sure, read the appropriate sections before making your selection.

☒ Life sciences ☐ Behavioural & social sciences ☐ Ecological, evolutionary & environmental sciences

For a reference copy of the document with all sections, see nature.com/documents/nr-reporting-summary-flat.pdf

Life sciences study design

All studies must disclose on these points even when the disclosure is negative.

Sample size	Data were repeated at least three (3) times
Data exclusions	No data were excluded from the analysis
Replication	All attempts at replication were successful
Randomization	No samples/organisms/participants were used in this study
Blinding	No samples/organisms/participants were used in this study, so no blinding was possible

Reporting for specific materials, systems and methods

We require information from authors about some types of materials, experimental systems and methods used in many studies. Here, indicate whether each material, system or method listed is relevant to your study. If you are not sure if a list item applies to your research, read the appropriate section before selecting a response.

Materials & experimental systems

n/a	Involved in the study	
☐	☒	Antibodies
☐	☒	Eukaryotic cell lines
☒	☐	Palaeontology and archaeology
☒	☐	Animals and other organisms
☒	☐	Clinical data
☒	☐	Dual use research of concern
☒	☐	Plants

Methods

n/a	Involved in the study	
☒	☐	ChIP-seq
☒	☐	Flow cytometry
☒	☐	MRI-based neuroimaging

Antibodies

Antibodies used	M1 anti-FLAG antibody (4E11, ATCC) was used for in vitro pull-down.
Validation	Validated by the provider and used in many published studies.

Eukaryotic cell lines

Policy information about cell lines and Sex and Gender in Research

Cell line source(s)	Parental HEK293SL and βarr1/2 KO cells were a gift from Dr Stephane Laporte (McGill University, Montreal, Quebec, Canada). HEK293T, Gαs KO and total Gα KO cells were a gift from Dr Asuka Inoue (Tohoku University, Sendai, Miyagi, Japan).
Authentication	No cell line authentication
Mycoplasma contamination	Cells were regularly tested for mycoplasma contamination (PCR Mycoplasma Detection kit, abm) and only micoplasm-free cells were used.
Commonly misidentified lines (See ICLAC register)	n/a
